# Septal venous channel perforation during left bundle branch area pacing: a prospective study

**DOI:** 10.1093/europace/euae124

**Published:** 2024-05-04

**Authors:** Anindya Ghosh, Anbarasan Sekar, Chenni S Sriram, Kothandam Sivakumar, Gaurav A Upadhyay, Ulhas M Pandurangi

**Affiliations:** Department of Cardiac Electrophysiology and Pacing, Arrhythmia Heart Failure Academy, The Madras Medical Mission, 4-A, Dr. JJ Nagar, Mogappair, Chennai, Tamil Nadu 600037, India; Department of Cardiac Electrophysiology and Pacing, Arrhythmia Heart Failure Academy, The Madras Medical Mission, 4-A, Dr. JJ Nagar, Mogappair, Chennai, Tamil Nadu 600037, India; Division of Cardiology, Sub-section of Electrophysiology, Children’s Hospital of Michigan and Detroit Medical Center, Detroit, MI, USA; Department of Pediatric Cardiology, Institute of Cardiovascular Diseases, The Madras Medical Mission, Chennai, Tamil Nadu, India; Center for Arrhythmia Care, Section of Cardiology, University of Chicago Pritzker School of Medicine, Chicago, IL, USA; Department of Cardiac Electrophysiology and Pacing, Arrhythmia Heart Failure Academy, The Madras Medical Mission, 4-A, Dr. JJ Nagar, Mogappair, Chennai, Tamil Nadu 600037, India

**Keywords:** Septal venous channel perforation, LBBAP, Coronary sinus tributary, Electrophysiological characteristics, Complications, Coronary sinus visualization

## Abstract

**Aims:**

To characterize the diagnosis, frequency, and procedural implications of septal venous channel perforation during left bundle branch area pacing (LBBAP).

**Methods and results:**

All consecutive patients undergoing LBBAP over an 8-month period were prospectively studied. During lead placement, obligatory septal contrast injection was performed twice, at initiation (implant entry zone) and at completion (fixation zone). An intuitive fluoroscopic schema using orthogonal views (left anterior oblique/right anterior oblique) and familiar landmarks is described. Using this, we resolved zonal distribution (I–VI) of lead position on the ventricular septum and its angulation (post-fixation angle *θ*). Subjects with and without septal venous channel perforation were compared. Sixty-one patients {male 57.3%, median age [interquartile range (IQR)] 69.5 [62.5–74.5] years} were enrolled. Septal venous channel perforation was observed in eight (13.1%) patients [male 28.5%, median age (IQR) 64 (50–75) years]. They had higher frequency of (i) right-sided implant (25% vs. 1.9%, *P* = 0.04), (ii) fixation in zone III at the mid-superior septum (75% vs. 28.3%, *P* = 0.04), (iii) steeper angle of fixation—median *θ* (IQR) [19 (10–30)° vs. 5 (4–19)°, *P* = 0.01], and (iv) longer median penetrated-lead length (IQR) [13 (10–14.8) vs. 10 (8.5–12.5) mm, *P* = 0.03]. Coronary sinus drainage of contrast was noted in five (62.5%) patients. Abnormal impedance drops during implantation (12.5% vs. 5.7%, *P* = NS) were not significantly different.

**Conclusion:**

When evaluated systematically, septal venous channel perforation may be encountered commonly after LBBAP. The fiducial reference framework described using fluoroscopic imaging identified salient associated findings. This may be addressed with lead repositioning to a more inferior location and is not associated with adverse consequence acutely or in early follow-up.

## Graphical Abstract

**Graphical Abstract:**
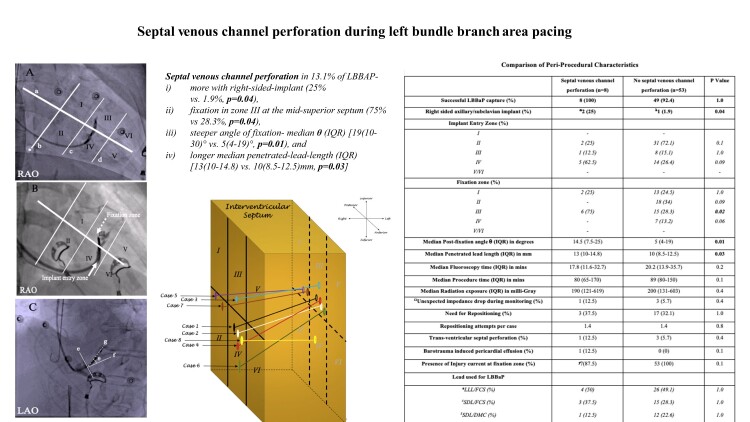

**What’s new?**

- Inadvertent perforation of the septal venous channels with or without additional drainage into a coronary sinus tributary during left bundle area pacing is not uncommon contrary to popular belief, and was seen and characterized in 13.1% of the study population.
- Additionally, the same did not significantly affect procedure outcomes when compared to those without this complication.

## Introduction

Procedural complications during left bundle branch area pacing (LBBAP) are reported in up to 20% of patients.^1^ Interventricular septal perforation is common with a prevalence of 0–14%.^1^ However, septal venous channel perforation is rarely reported (<1%) and is not as well characterized.^2–5^ We sought to systematically analyse the frequency, procedural characteristics, and implications associated with septal venous channel perforation in patients undergoing routine LBBAP.

## Methods

This was a prospective study of consecutive patients undergoing LBBAP for guideline-based pacing indications between June 2023 and January 2024 at a single tertiary-care centre.^6^ Patients with history of contrast allergy and at risk for contrast-induced nephropathy due to underlying chronic kidney disease were excluded. Demographic, clinical, and procedural characteristics were compared in patients with and without septal venous channel perforation. The site’s institutional review board approved this study. Written informed consent after full disclosure was obtained from all patients.

### Left bundle branch area pacing protocol

The study included both lumen-less and stylet-driven leads (SDLs) with the vendor-specific corresponding sheath delivery sets. The following combinations were used: Medtronic C315HIS delivery sheath and Medtronic SelectSecure 3830 lead (Minneapolis, MN, USA); Biotronik Selectra 3D sheaths (55/59 and 40/39) and the Biotronik Solia S60 lead (Berlin, Germany); the Abbott Agilis HisPro™ delivery sheath and the Abbott Tendril STS 2088 lead (Sylmar, CA); and Boston Scientific Site Selective Pacing Catheter (SSPC) and Ingevity 4197–59 lead.

Our implantation techniques for SDLs are previously published.^7^ Lumen-less lead (LLL) implant was performed as per established protocol.^8^ Unipolar impedance measurements were documented at initiation of screwing-in (implant entry zone) and at completion of screwing (fixation zone) for all patients. The details pertaining to these zones are elaborated below. For the SDLs, impedance was also continuously measured throughout the course of lead placement. In case of LLL, impedance was monitored at periodic intervals. Lead perforation across the interventricular septum (unexpected drop in impedance) was suspected in case of an acute impedance drop of >200 Ω while implanting SDLs and a drop to an absolute value < 450 Ω (at any time) in case of LLL.^9,10^

### Septal angiogram during left bundle branch area pacing and fluoroscopic analysis

Standard fluoroscopic projections including right anterior oblique (RAO 20–25°) and left anterior oblique (LAO 30–35°) views were obtained interchangeably using the anteroposterior camera. The delivery sheath was positioned over the right ventricular (RV) septal endocardial surface in the RAO view. The appropriate site was selected based on the paced ECG morphology.^7^ The perpendicular orientation of the sheath with respect to the septum was then adjusted and ascertained in LAO projection. Subsequently, 10 mL of radio-opaque contrast (Visipaque; 1:1 dilution) was hand-injected into the irrigation port of the delivery catheter. Fluoroscopic analysis was performed in real time and fluoroscopy duration was adjusted depending on visualization of any septal venous channel or distal coronary sinus tributary. The criteria for the latter are separately elaborated in a sub-section below.

As per our pre-ordained study protocol, the lead was then delivered into the septum at the same site irrespective of the angiographic findings. The depth of septal penetration was primarily guided by (i) typical morphology of fixation beats, and specifically for SDLs parameters such as (ii) continuous unipolar tip-paced ECG morphology, and (iii) unipolar impedance trends.^9^ At this point, a follow-up contrast angiogram was performed in the same view as described above. Angiogram was performed with delivery sheath adequately abutting the septum to optimize visualization of coronary venous channels. This manoeuver was also likely to eliminate any false positive findings secondary to staining of endocardial recesses or trabeculations. Additional orthogonal projection (RAO) was left at the discretion of the implanting physician. Lead capture thresholds, sensing parameters, and local injury current on electrogram were recorded at the onset and completion of screwing. Pacing impedance trends were measured as previously described.

Our study protocol also allowed for refixation/repositioning of the leads based on any unsatisfactory parameters/procedural complications at the discretion of the physician after attempt(s) at fixation. At the end of the procedure, we analysed our success rate of LBBAP capture based on established criteria. Additional parameters including procedural/fluoroscopy times, median radiation exposure, and number of repositioning attempts were also analysed.

### Definition and adjudication of septal venous channels

Patients with septal venous channel perforation were internally adjudicated fluoroscopically by our institutional physicians (U.M.P. and A.G.) based on pre-specified criteria as enunciated below. These were subsequently validated (*Supplementary Videos 1–8*) or rejected (*Supplementary Video 9*) by two blinded external reviewers (C.S.S. and G.A.U.). Validation required independent agreement by both the reviewers.

A septal venous channel was defined as an area of transient septal opacification (*Supplementary Video 5*) (distinguishable from the usual persistent myocardial staining; *Supplementary Video 10*) characterized by complete washout of radio-opaque contrast material within three cardiac cycles at a fluoroscopic frame-rate of 7.5 per second. Any additional drainage of contrast into a coronary venous sinus (CS) tributary was also corroborated (*Supplementary Video 1*).

### Post-implant analysis of left bundle branch area pacing lead position on stored fluoroscopic images

This was performed internally at our institution by one physician (A.G.) and validated by the second (U.M.P.) who was not blinded. One blinded external reviewer (C.S.S.) was privy to our measurements and helped with remote electronic adjudication. Disagreements were resolved by consensus.

**Figure 1:**
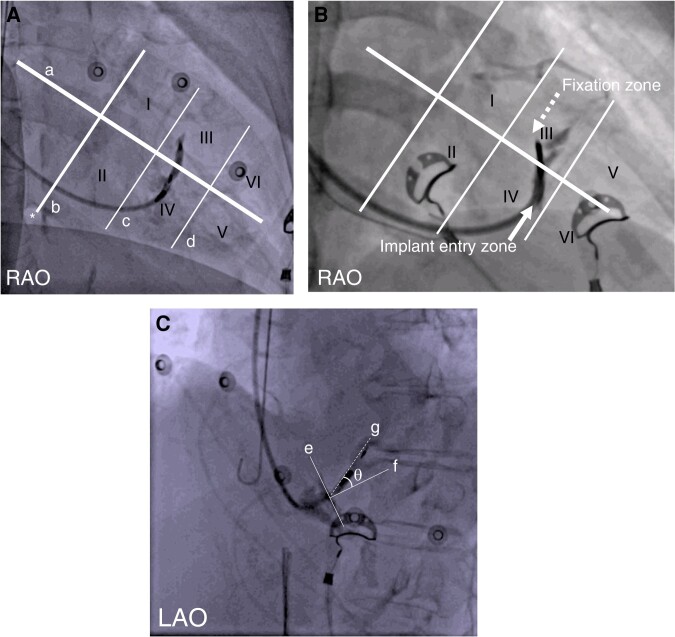
Fluoroscopic fiducial reference framework. (*A*) Delineation of zones on right ventricular endocardial aspect in the right anterior oblique (RAO) view. (*B*) Denotation of implant entry and fixation zones. (*C*) Measurement of post-fixation angle (*θ*) in left anterior oblique (LAO) view. Please refer to Methods for additional details.

The RV endocardial aspect as seen in the RAO view was defined and divided into six equidistant zones as shown in *Figure 1A*. A straight line (Line a) was first inscribed along the long axis of the heart from the apex to base. The inferior radiolucent area (labelled as *) within the cardiac silhouette in the RAO view was identified as the fluoroscopic landmark for a prominent epicardial fat pad in the left atrioventricular groove in close proximity to the mouth of the coronary sinus.^11^ A perpendicular line (Line b) to the long axis of the heart was drawn from this radiolucent area. The cardiac silhouette between Line b and the apex was divided into six equidistant zones (I–VI) by two additional lines (Lines c and d) drawn perpendicular to Line a (parallel to Line b). Based on the above defined paradigm, we separately delineated the implant entry zone (prior to screwing-in) as well as final fixation zone for each patient (*Figure 1B*).

The post-fixation angle (*θ*) was measured by online protractor on the LAO view as shown in *Figure 1C*. The plane of the septum was identified based on contrast delineation and inscribed by Line e. Line f was inscribed perpendicular to Line e (at the point of lead entry). Line g was then drawn along the axis of the lead. The angle *θ* was measured between Lines f and g.

The implant entry zone/fixation zone (RAO view) and post-fixation angle *θ* were compared in patients with and without septal venous channel perforation. Lead penetration length was defined as the total length of the lead within the fluoroscopically defined septum in LAO view (length of Line g in *Figure 1C*).

### Pre-discharge echocardiographic imaging

All patients initially deemed to have septal venous channel perforation underwent trans-thoracic echocardiogram the following day after the procedure. They were specifically evaluated for any intramural haematoma, pericardial effusion, and new regional wall motion abnormalities. Cross-sectional imaging was not part of the study protocol.

### Post-implant follow-up

At a 1-month follow-up, we collated all relevant device parameters including impedance, sensing, as well as pacing thresholds. Twelve-lead electrocardiographic analysis of paced QRS morphology, axis, and duration was also ascertained.

### Statistical analysis

IBM SPSS Ver 23.0 software (SPSS Inc., Chicago, IL, USA) was used for statistical analysis. The categorical variables were expressed as frequencies/percentages. Continuous variables were represented as median with the 25th–75th interquartile range (IQR). Mann–Whitney *U* test was used to compare continuous variables. Fisher’s exact test was used for analysing categorical data. A *P* value ≤ 0.05 was considered to be statistically significant.

## Results

Left bundle branch area pacing was performed in 62 consecutive patients for guideline-directed indications in the treatment of bradyarrhythmia or for cardiac resynchronization therapy. None of our patients were prescribed oral anticoagulants before or after the procedure. One patient was excluded from septal contrast injection due to the presence of chronic kidney disease and risk for contrast-induced nephropathy. Left bundle branch area pacing with septal contrast injection was performed in the remaining 61 patients [male 57.3%, median age (IQR) 69.5 (62.5–74.5) years, median left ventricular ejection fraction or LVEF % (IQR) 55 (45–57.5)] with additional clinical characteristics as depicted in *Table 1*. All patients, except one 16-year-old male, were adults.

**Table 1:** Demographic and clinical characteristics of patients with LBBAP (*n* = 61)

Median age (IQR) in years	69.5 (62.5–74.5)
Male sex (%)	35 (57.3)
Indications for pacing	
Sick sinus syndrome	6 (9.8)
1st degree AV block with/without bundle branch block (%)	3 (4.9)
2nd degree AV block (%)	10 (16.4)
2:1 AV block (%)	4 (6.6)
Complete AV block (%)	31 (50.8)
Class I CRT indication (%)	6 (9.8)
As a part of AV junction ablation (%)	1 (1.6)
Ischaemic heart disease (%)	29 (47.5)
Type 2 diabetes mellitus (%)	41 (67.2)
Hypertension (%)	39 (63.9)
Median QRS duration (IQR) in ms	120 (100–130)
Median LVEF (IQR) in %	55 (45–57.5)
LVEF ≤ 40% (%)	11 (18)
Cardiac implantable electronic device	
Dual chamber pacemaker (%)	51 (83.6)
Biventricular pacemaker (%)	1 (1.6)
Dual chamber AICD (%)	9 (14.8)
Lead used for LBBAP	
LLL/FCS (%)^a^	30 (49.2)
SDL/FCS (%)^b^	18 (29.5)
SDL/DMC (%)^c^	13 (21.3)

LLL, lumen-less lead; FCS, fixed curve sheath; SDL, stylet-driven lead; DMC, deflectable mapping catheter.

^a^LLL/FCS—Proprietary Medtronic SelectSecure 3830 lead/C315His sheath.

^b^SDL/FCS—Proprietary Biotronik Solia S60 lead/Selectra 3D sheath or Proprietary Boston Scientific Ingevity lead/Site Selective Pacing Catheter (SSPC).

^c^SDL/DMC—Proprietary Abbott Tendril STS 2088 lead/Agilis HisPro.

**Figure 2:**
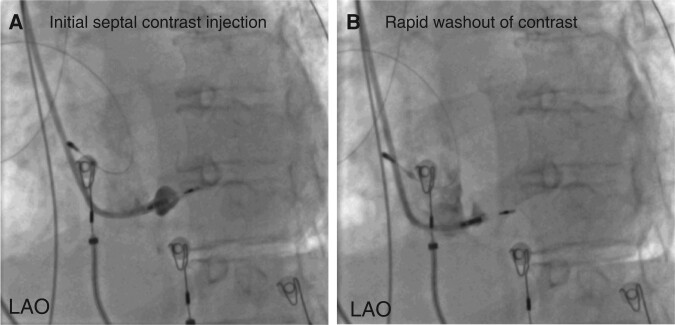
Example of venous channel perforation without drainage into CS. Septal venous channel perforation (protocol-defined) without additional drainage into coronary sinus tributary.

**Figure 3:**
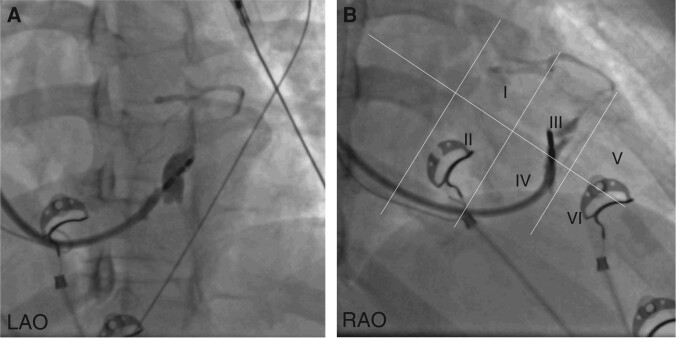
Example of venous channel perforation and subsequent drainage into CS with an acute post-fixation angle *θ*. Septal venous channel perforation (protocol-defined) with additional drainage into coronary sinus tributary.

**Figure 4:**
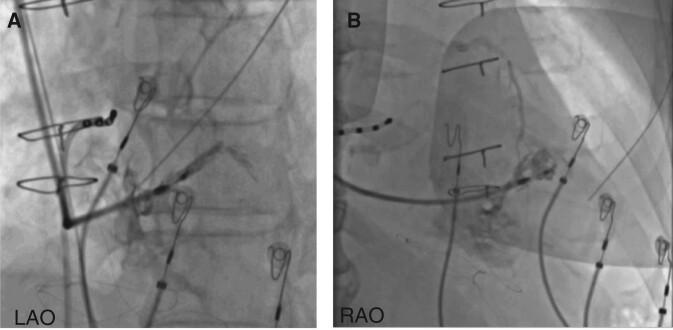
Example of venous channel perforation and subsequent drainage into CS with post-fixation angle *θ* = 0. Septal venous channel perforation (protocol-defined) with additional drainage into septal perforator tributaries of anterior interventricular vein and middle cardiac vein and subsequent visualization of coronary sinus.

**Figure 5:**
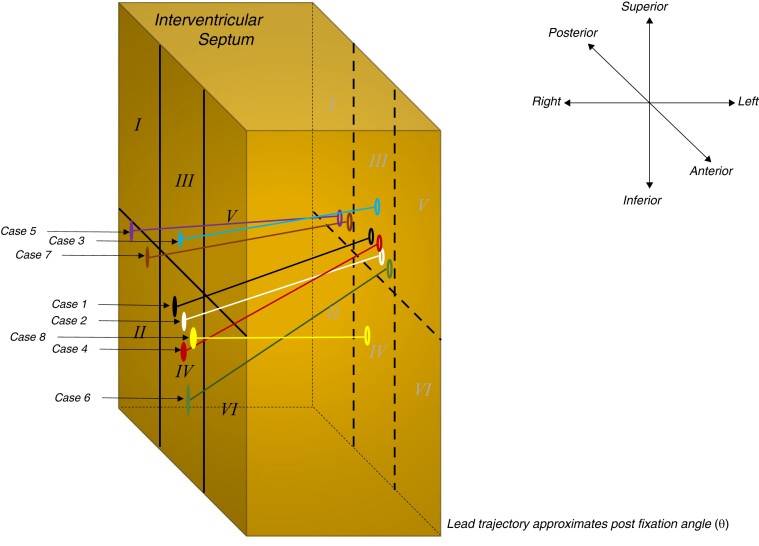
Schematic diagram showing lead orientation inside the septum for the eight cases with septal venous channel perforation.

Septal venous channel perforation (*Figure 2*) as defined in the study protocol was observed in 8 (13.1%) patients [male 37.5%, median age (IQR) 64 (50–75) years, median LVEF % (IQR) 55 (40–60)]. Venous channels were noted at the implant entry zone (at initial contrast injection before lead delivery) in two subjects, and at the fixation zone (injection after lead delivery) in the remaining six patients. None had simultaneous findings on both angiograms. Additional drainage into a CS tributary (*Figures 3* and *4*) was noted in five patients, at the implant entry zone/initial contrast injection for one patient (*Supplementary Video*/Case 1) and at the fixation zone/follow-up contrast injection for the remainder (*n* = 4; *Supplementary Videos*/Cases 3, 4, 6, and 8). A schematic diagram with approximate trajectories of the lead/sheath in cases of septal venous channel perforation is presented in *Figure 5*.

A comparison of cases with and without septal venous channel perforation with respect to baseline, peri-procedural, and follow-up lead parameters and ECG characteristics are highlighted in *Tables 2*–*4*, respectively. There were no significant differences in demographic or clinical characteristics. There were notable differences between the groups with respect to peri-procedural or implant characteristics (*Table 3*). Patients with septal venous channel perforation demonstrated a higher frequency of (i) right-sided device implant (25% vs. 1.9%, *P* = 0.04), (ii) implant in fixation zone III (superior-mid septum) (75% vs. 28.3%, *P* = 0.02), (iii) more acute median post-fixation angle *θ* (IQR) [14.5 (7.5–25)° vs. 5 (4–19)°, *P* = 0.01], and (iv) a longer median penetrated-lead length (IQR) [13 (10–14.8) vs. 10 (8.5–12.5) mm, *P* = 0.03]. Other parameters including any unexpected drop in impedance while implanting the lead (12.5 vs. 5.7%, *P* = NS) did not reach statistical significance.

**Table 2:** Comparison of baseline characteristics

	Septal venous channel perforation (*n* = 8)	No septal venous channel perforation (*n* = 53)	*P* value
Median age (IQR) in years	64 (50–75)	70 (62–74)	0.059
Male sex (%)	3 (37.5)	19 (35.8)	0.6
Ischaemic heart disease (%)	4 (50)	25 (58.1)	0.6
Type 2 diabetes mellitus (%)	6 (75)	35 (66)	1.0
Hypertension (%)	4 (50)	35 (66)	0.6
Median QRS duration (IQR) in ms	120 (110–130)	120 (100–130)	0.3
Median LVEF (IQR) in %	55 (40–60)	55 (40–55)	0.4
Median septal thickness (IQR) in mm	10 (8–11)	10 (9–12)	0.5

**Table 3:** Comparison of peri-procedural characteristics

	Septal venous channel perforation (*n* = 8)	No septal venous channel perforation (*n* = 53)	*P* value
Successful LBBAP capture (%)	8 (100)	49 (92.4)	1.0
Right-sided axillary/subclavian implant (%)	2 (25)^a^	1 (1.9)^b^	0.04
Implant entry zone (%)			
I	—	—	
II	2 (25)	31 (72.1)	0.1
III	1 (12.5)	8 (15.1)	1.0
IV	5 (62.5)	14 (26.4)	0.09
V/VI	—	—	—
Fixation zone (%)			
I	2 (25)	13 (24.5)	1.0
II	—	18 (34)	0.09
III	6 (75)	15 (28.3)	0.02
IV	—	7 (13.2)	0.06
V/VI	—	—	
Median post-fixation angle *θ* (IQR) in degrees	14.5 (7.5–25)	5 (4–19)	0.01
Median penetrated-lead length (IQR) in mm	13 (10–14.8)	10 (8.5–12.5)	0.03
Median fluoroscopy time (IQR) in min	17.8 (11.6–32.7)	20.2 (13.9–35.7)	0.2
Median procedure time (IQR) in min	80 (65–170)	89 (80–150)	0.1
Median radiation exposure (IQR) in milli-Gray	190 (121–619)	200 (131–603)	0.4
Unexpected impedance drop during monitoring (%)^c^	1 (12.5)	3 (5.7)	0.4
Need for repositioning (%)	3 (37.5)	17 (32.1)	1.0
Repositioning attempts per case	1.4	1.4	0.8
Trans-ventricular septal perforation (%)	1 (12.5)	3 (5.7)	0.4
Barotrauma induced pericardial effusion (%)	1 (12.5)	0 (0)	0.1
Presence of injury current at fixation zone (%)	7 (87.5)^d^	53 (100)	0.1
Lead used for LBBAP			
LLL/FCS (%)^e^	4 (50)	26 (49.1)	1.0
SDL/FCS (%)^f^	3 (37.5)	15 (28.3)	1.0
SDL/DMC (%)^g^	1 (12.5)	12 (22.6)	1.0

LLL, lumen-less lead; FCS, fixed curve sheath; SDL, stylet-driven lead; DMC, deflectable mapping catheter.

^a^Right-sided implant was necessitated in two female patients with left-sided breast carcinoma with surgical mastectomy and need for local radiation therapy.

^b^One patient underwent right-sided implant because of chronic subtotal occlusion of left subclavian vein.

^c^Unexpected impedance drop as per monitoring during lead placement as described in Methods.

^d^Injury current was lost in one patient following myocardial septal perforation.

^e^LLL/FCS—Proprietary Medtronic SelectSecure 3830 lead/C315His sheath.

^f^SDL/FCS—Proprietary Biotronik Solia S60 lead/Selectra 3D sheath or Proprietary Boston Scientific Ingevity lead/Site Selective Pacing Catheter (SSPC).

^g^SDL/DMC—Proprietary Abbott Tendril STS 2088 lead/Agilis HisPro.

**Table 4:** Comparison of electrocardiographic and lead parameters based on final lead position

Septal contrast angiogram	Lead through septal venous channel (*n* = 5)^a^	Lead not through septal venous channel (*n* = 56)^b^	*P* value
Median pacing impedance (IQR) in ohms			
At implant^c^	666 (650–760)	649 (640–720)	0.8
At 1-month follow-up	631 (526–650)	650 (610–720)	1.0
Median capture threshold-unipolar tip (IQR) in volts at 0.4 ms PW			
At implant^c^	0.6 (0.5–0.7)	0.8 (0.6–0.9)	0.7
At 1-month follow-up	0.5 (0.3–0.7)	0.8 (0.7–0.9)	0.5
Median sensed R waves (IQR) in millivolts			
At implant^c^	8.4 (7.6–9.3)	9.7 (8.8–10.3)	0.6
At 1-month follow-up	8.1 (7.8–9.5)	9.1 (8.3–10.5)	0.6
Median paced QRS duration (IQR) in ms			
At implant^c^	110 (0)	110 (100–120)	0.9
At 1-month follow-up	110 (90–115)	110 (110–130)	0.8

^a^Only 5/8 patients in whom septal venous channel was assessed required no repositioning of the lead. 3/7 patients needed lead repositioning to a new location devoid of a venous channel.

^b^The 3/7 patients that needed lead repositioning as noted above were added to the 42 patients without demonstrable venous channel.

^c^At implant values were obtained after lead fixation.

Salient characteristics of patients with septal venous channel perforation (*n* = 8) are described in *Table 5*. As previously noted, septal angiogram was performed at implant entry zone (before screwing-in) and at fixation zone (after screwing-in). Venous channel was accessed at the first attempt in 7/8 (87.5%) subjects. Of these seven, lead repositioning was needed in three patients due to absence of confirmation of left bundle branch (LBB) capture (*n* = 1; Case 1), trans-ventricular septal myocardial perforation with loss of capture (*n* = 1; Case 5 with documentation of perforation in *Supplementary Video 5*), and inadvertent barotrauma/volutrauma-induced pericardial effusion related to contrast injection (*n* = 1; Case/*Supplementary Videos 6A*–*6C*), respectively. Venous channel was accessed in 1/8 (12.5%) patient at the 2nd attempt following lack of LBB capture at the initial attempt. At the end of the procedure, all eight patients (five subjects with lead through septal venous channel and three that needed repositioning after assessing septal venous channel) had achieved successful LBBAP. In one instance (Case 5/*Figure 2*/*Supplementary Video 5*), the images may be subjectively suboptimal in showcasing the intended degree of difference between septal venous channel and pouch of endocardial tissue. The external reviewers (C.S.S. and G.A.U.) adjudicated this as the former based on overall strength of evidence.

**Table 5:** Salient characteristics of patients with septal venous channel perforation

Cases	Age (years)	Sex	Primary cardiac diagnosis	Proprietary lead/sheath combo	Right vs. left-sided implant	Septal venous channel perforation	Septal venous channel demonstration	Post-fixation angle (*θ*)	LBBA capture after fixation	Ventricular septal perforation	Barotrauma induced pericardial effusion	Lead repositioning^a^
1	64	F	CHB	SDL/DMC	Left	At 1st attempt	Follow-up angiogram	20	No	No	No	Yes^λ^
2	77	F	CHB	LLL/FCS	Right	At 1st attempt	Initial angiogram	19	Yes	No	No	No
3	50	F	SSS	SDL/FCS	Left	At 1st attempt	Follow-up angiogram	10	Yes	No	No	No
4	16	M	Repaired DORV VSD PS with CHB	LLL/FCS	Left	At 1st attempt	Follow-up angiogram	30	Yes	No	No	No
5	75	F	CHB	LLL/FCS	Left	At 2nd attempt	Follow-up angiogram	5	No	Yes^b^	No	Yes^α^
6	65	F	CHB	SDL/DMC	Right	At 1st attempt	Initial angiogram	32	Yes	No	Yes^c^	Yes
7	58	M	Dilated cardiomyopathy	LLL/FCS	Left	At 1st attempt	Follow-up angiogram	10	Yes	No	No	No
8	60	M	Ischaemic cardiomyopathy	SDL/FCS	Left	At 1st attempt	Initial angiogram	0	Yes	No	No	No

SDL/DMC—Proprietary Abbott Tendril STS 2088 lead/Agilis HisPro, LLL/FCS—Proprietary Medtronic SelectSecure 3830 lead/C315His sheath, SDL/FCS—Proprietary Biotronik Solia S60 lead/Selectra 3D sheath or Proprietary Boston Scientific Ingevity lead/Site Selective Pacing Catheter (SSPC).

CHB, complete heart block; SSS, sick sinus syndrome; DORV, double outlet right ventricle; VSD, ventricular septal defect; PS, pulmonic stenosis; SDL, stylet-driven lead; DMC, deflectable mapping catheter; LLL, lumen-less lead; FCS, fixed curve sheath.

^a^Lead repositioning was needed due to lack of LBBA capture following fixation (^λ^Case 1) and due to ventricular septal perforation with loss of capture (^α^Case 5).

^b^Trans-ventricular septal perforation was noted in Case 5 fluoroscopically along with loss of capture.

^c^Contrast injection resulted in inadvertent barotrauma induced rupture of epicardial CS tributaries with mild pericardial effusion (*Supplementary Video 6*). The lead position was changed and subsequent successful left bundle branch capture could be obtained.

An exact diagnosis of chest pain directly related to venous channel perforation remains practically difficult in our cohort for the following reasons. Some amount of chest discomfort and incisional site pain is already anticipated post-procedure and therefore empirically/prophylactically treated with analgesics. Nevertheless, severe sustained or anginal chest pain or that unresponsive to the usual analgesics was not noted in any of our eight patients. None of our patients needed any anti-anginal medications for relief of chest pain.

In this study, pre-discharge echocardiogram was only selectively performed in the eight patients already diagnosed with a septal venous channel perforation. In those, there was no echocardiographic/colour Doppler evidence of a fistula between the venous system and the right ventricle. No intramural haematoma or new regional wall motion abnormalities were noted. A residual small pericardial effusion was noted in the singular patient (1/8 or 12.5%; Case 6) who incurred barotrauma as noted above. This resolved in follow-up echocardiogram performed at 4 weeks after implant.

## Discussion

Left bundle branch area pacing is increasingly recognized as a modality of conduction system pacing to optimize physiological cardiac resynchronization.^12,13^ With growing overall world experience, there has been an evolving learning curve with respect to the risk of intraprocedural complications and management. This prospective single-centre sentinel exploratory study was designed in this background to address a specific lacuna in literature, namely coronary venous channel perforation. Our main objective was to formalize the identification of this hitherto under-reported entity, characterize its frequency, and appreciate any ensuing procedural implications. The methodology included comparison with subjects without the above finding and identify associated relevant clinical as well as electrophysiological and anatomical variables.

Obligatory septal contrast injection at initiation of screwing-in the lead and pursuant its fixation revealed that a coronary venous channel was perforated in a significant minority (8/61 or 13.1%). Nevertheless, it remained conducive to lead retention and successful LBBAP in the majority (62.5%). It entailed a similar requirement for lead repositioning (37.5 vs. 32.1%, *P* = NS) and even if encountered during procedure, there was no ultimate adverse impact on the requisite outcome of successful LBBAP (100 vs. 95.2%, *P* = NS). No demographic or baseline clinical characteristics were over/under-represented. In particular, frequent or continuous monitoring of unipolar lead tip impedance during implant did not clue us in towards the diagnosis of venous channel. The procedure or fluoroscopy time, radiation exposure, lead type (lumen less vs. stylet driven), sheath type, and post-fixation lead parameters (at implant and 1-month follow-up) were also similar between the groups.

Overall, five complications were reported (two in cases with septal venous channel perforation and three without it), representing a directionally relevant but clinically insignificant trend (25 vs. 5.7, *P* = NS). These included (i) interventricular septal myocardial perforation (12.5 vs. 5.7%, *P* = NS; Case/*Supplementary Video 5*) and (ii) singular unique complication of inadvertent barotrauma induced by the contrast injection leading to pericardial effusion (12.5 vs. 0%, *P* = NS; Case/*Supplementary Videos 6A*–*6C*). The latter was deemed to be a clinically significant, hitherto unreported complication. Fortunately, it was not associated with any acute haemodynamic compromise or follow-up sequelae. There were specifically no instances of inadvertent arterial entry during contrast injection. But the authors at present cannot authoritatively state that this is unlikely to happen because of these smaller numbers.

Our study protocol dictated that a pre-discharge echocardiogram be performed exclusively in patients with demonstrable septal venous channel perforation. However, based on our study results, we are in the process of instituting a policy of performing a pre-discharge routine echocardiogram targeted towards the diagnosis of septal haematoma and pericardial effusion in all patients who underwent LBBAP. In those who were previously prescribed anticoagulation, its resumption after the procedure must be balanced not only in the context of clotting risk vs. pocket haematoma, but also factoring any such complications noted above. At present, this is left at the discretion of the implanting physician.

Most implanting physicians typically inject 2–5 mL of radio-opaque dye for performing septal contrast angiogram. The larger than usual volume of contrast (10 mL, 1:1 dilution) injection employed in this study was deliberately chosen as part of our study protocol. Herein, our primary objective was to improve the sensitivity/optimal fluoroscopic visualization of coronary venous channels including any CS tributaries. In the same regard, evidence of rapid washout of a larger volume of contrast also increased our confidence to robustly adjudicate the above finding. Whether a larger volume of contrast injection into a septal coronary venous channel accentuates the risk of barotrauma/volutrauma/pericardial effusion via pressure transmission to the connecting CS tributaries is a clinically valid question. It is deemed to be a topic for further investigation.

The practicing electrophysiologist is privy to contextualizing the three-dimensionality of the lead implant on the ventricular septum in terms of orthogonal views profiling it along its length (RAO view) and width (LAO view). The authors have leveraged and expanded on this concept along with use of some familiar landmarks to design a schema (*Figure 1*). Herein, the ventricular septal aspect is divided into six equidistant zones (RAO view) and the angular orientation/length of the lead traversing the septum (LAO view) is also described. We hope that this nascent proposal will be adopted as an intuitive and practical tool while referencing lead position (implant and fixation zone) and its directionality (post-implant angle *θ*) in LBBAP.

Analytical representation of our data within the above framework yielded tangible and significant findings in cases with septal venous perforation. These included (i) over-representation of fixation zone III (superior aspect of the middle one-third of ventricular septum), (ii) a more acute angulation of lead with respect to, and (iii) longer penetrated-lead length within the septum. These paradigms are easily conceptualized as by-products of an oblique (as well as expected superior) trajectory of the lead traversing the perforated venous channel. The higher frequency of right subclavian/axillary implant in cases with septal venous channel perforation (25% vs. 1.9%, *P* = 0.04) may represent a statistical artefact of small numbers. Any anatomical hypothesis pertaining to lead orientation and directionality warrants further validation and therefore it is prudent to refrain from additional speculation in the interim.

Unipolar impedance monitoring (continuous for SDL and periodic for LLL) remained an integral part of our procedure as enunciated in the methods. During the course of implant, there were no unexpected changes in impedance (i.e. exceeding the described cut-offs in methods) for the majority [7/8 vs. 50/53 (i.e. 87.5 vs. 94.3%), *P* = NS].

**Figure 6:**
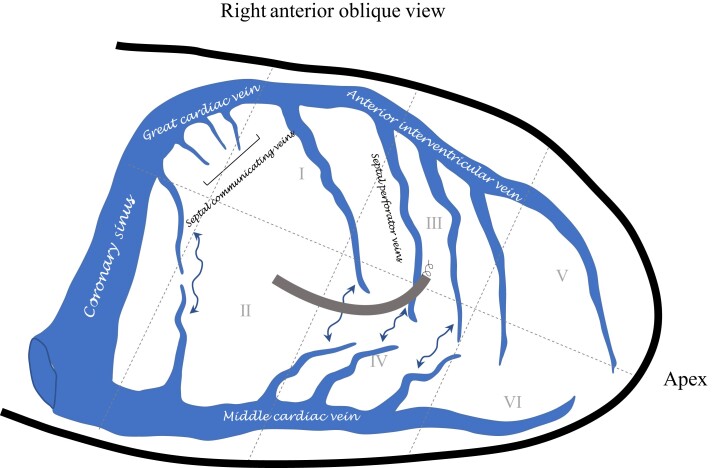
Schematic diagram of interventricular septal venous network. Septal perforating venous network representing venous channels draining the interventricular septum.

The coronary venous channels discussed here are expected to represent the branching and inter-communicating ventricular septal venous perforator network forming the tributaries of the anterior interventricular and middle cardiac veins (*Figure 6*). These predominantly drain the upper two-thirds and lower one-third of the ventricular septum, respectively.^2,14–16^ Demonstration of additional venous drainage in 5/8 (62.5%) of our cases into a prominent coronary sinus tributary lends further credence to this notion. Based on the attempted access point on the septum for LBBAP and the natural orientation of the lead from the superior vena cava, the perforating venous network tributaries of the anterior interventricular vein are more likely to be injured during a venous channel perforation.

Given the profusion of this venous septal network and increasing traction in the adoption of LBBAP, accessing them should simply be a matter of statistical probability. Inadvertent perforation is probably under-recognized by the implanting physicians, possibly due to lack of systemic use of septal venous angiograms, lack of standardized definitions, and probably because of paucity of any adverse associated procedural outcomes. Ventricular myocardial capture through a venous structure is the central paradigm for coronary sinus lead implant and thus accessing this system is not a novel concept. Coronary venous channels within the ventricular septum may render a similar template for CSP.

The authors continue to advocate caution and reader discretion while interpreting these salient but nascent results. The primary aim here is to initiate a nuanced discussion, engender further research, and arrive at an erudite understanding on this subject. In particular, it is imperative to ascertain the exact clinical significance of such venous channels. Our small sentinel study is not a guarantor of its overall benign nature. Technical limitations pertaining to angle of the lead and depth of the sheath also require further refinement in future study.

This study does raise the spectre of safety vs. practical utility in accurately delineating septal venous channel perforation by injecting copious amount of contrast. Strictly speaking, the anatomical finding itself portended no adverse implant related electrophysiological parameters/outcomes thereby limiting the relevance/applicability of our protocol. Thus, the authors do not recommend such contemporaneous replication outside the context of research. Rather, the focus should aptly rest on using a smaller amount of contrast to demarcate the septum and facilitate the implant as needed. To surmise, although we showcase interesting anatomical insights, neither is our methodology deemed risk-free nor is it utilitarian.

### Limitations

Our prospective study is limited by its single-centre design and small numbers. The proposed definitional terminology for coronary venous channel perforation and fiducial fluoroscopic schemata describing lead position or orientation may be considered arbitrary. Therefore, it warrants further validation. Although unipolar lead impedance was frequently or continuously monitored during the implant for any unexpected drop, the assessment in our study of these findings was largely qualitative, and further quantitative assessment of impedance may reveal small but significant changes. As per our study protocol, coronary venous channel perforation could only be identified if the lead was inside its lumen. Perforation of a channel with lead exiting its lumen and landing in the adjacent myocardium would not be diagnosed. Therefore, our study has the potential to under-estimate the frequency of this unique finding. The authors also acknowledge practical limitations associated with identifying septal venous channels vs. right ventricular endocardial pouches in some cases (Case 5).

There are certain shortcomings with regards to blinding. Internal physicians who were also implanters were not blinded by default. The fluoroscopic views were generalized and not standardized individually, a practice similar to other centres. All fluoroscopic measurements were performed internally and validated by consensus. There was only limited external online supervision of this process by one blinded reviewer (C.S.S.). The two blinded external reviewers (C.S.S. and G.A.U.) only performed selective *post hoc* independent adjudication (*n* = 8) or rejection (*n* = 1) of possible coronary venous channel perforation. This was pursuant to these deemed positive findings showcased to them by the internal team (U.M.P. and A.G.).

Haemodynamic data collection, specifically right atrial mean or right-sided filling pressures that may have an impact on the size and distensibility of coronary venous channels, was not part of our study protocol. The use of different vendor-specific proprietary tools for LBBAP was dictated by local availability of hardware and technical support on the day of implant as well as the discretion of the implanting physician. With that noted, this was not an outcome determining variable. Pre-discharge echocardiogram was not performed universally, but limited to those with coronary venous channel perforation. Corroboration of the latter findings via post-implant high resolution cardiac computerized tomography scans was also not utilized.

## Conclusion

Inadvertent perforation of the septal venous channels with or without additional drainage into a coronary sinus tributary was plausibly identified with the meticulous use of septal contrast injection in a sizable minority (13.1%) of patients who underwent LBBAP. This maay be addressed with lead repositioning to a more inferior location. However, there may be practical issues limiting its accurate delineation in some instances. The fiducial reference framework described using fluoroscopic imaging identified salient associated findings. The acute/follow-up success rates and complications were comparable in subjects with and without septal venous channel perforation. The findings reported in this prospective single-centre study merit additional scrutiny in a larger population.

## Supplementary Material

euae124_Supplementary_Data

## Data Availability

The data that support the findings of this study are available from the corresponding author upon reasonable request.
